# Practical Skills en route to Professionalism

**DOI:** 10.3205/zma001065

**Published:** 2016-08-15

**Authors:** Kai P. Schnabel, Christoph Stosch

**Affiliations:** 1 Universität Bern, Institut für medizinische Lehre, Abteilung für Unterricht und Medien, Bern, Schweiz; 2 Universität zu Köln, Medizinische Fakultät, Referat für Lehre, Studium & Studienreform, Kölner Interprofessionelle Sklls Labs (KISS), Köln, Deutschland

## Foreword and annotations

**Figure 1:**
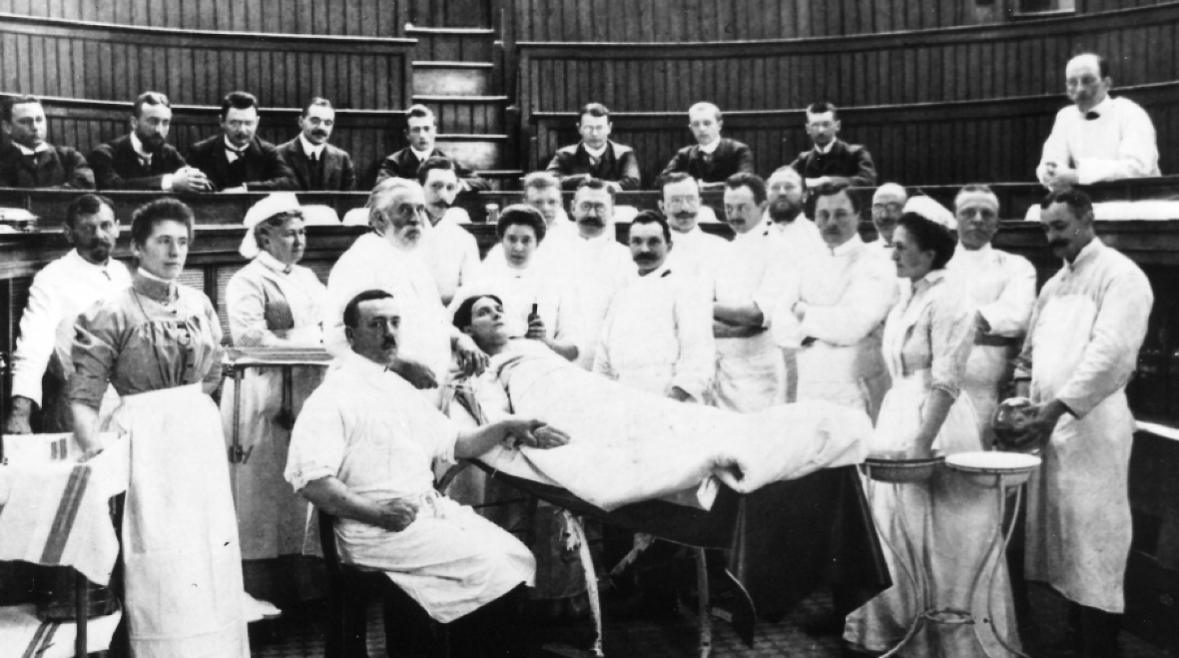
Prof. Ernst v. Bergmann, Demonstration operation, Ziegelstrasse, Berlin around the turn of the century.

The acquisition of practical skills has always been in the shadows of medical education. Until about 100 years ago, the pragmatic on model-oriented education was at the forefront and the teachers relied largely, on that the apprentices could require the needed practical skills through observation of the experts with the following practice on patients (see Figure 1 (Fig. 1)). The chair and his clinic, at which the apprentices are employed, guarantee the qualitative mediation of the practical skills.

Publications about practical skills are found in the field of training [1]. Since the ‘90s, practical skills during education have earned internationally an important value [2]. Simulations are also progressively winning value in the acquisition of practical skills and are in several areas being considered superior to traditional training methods [3]. Where once patients as training objects served, the role can often be taken now by the simulators or somewhat prepare the students technically better than the plain reading from textbooks and manuals. This doesn’t mean that they will not continue to train on the patients. Just like in the aviation, in which they have flight simulators to prepare the apprentices and assure qualitative outcomes, is now also imaginable in the fields of medicine. Practical procedures like blood taking or lumbar puncturing are being practiced in a safe environment under supervision on a model, before they are executed on live patients.

Since over 20 years, starting out in the fields of surgery, the OSCE (Objective Structured Clinical Examination) was established to inspect and assure practical skills [4]. Not only to certify but also to recertify in a way of uninterrupted quality assurance.

Consistent standardization through testing of practical skills and also the test-statistical requirements for the inspection (validity, objectivity and reliability) has called forth worldwide a variety of learn-goal catalogs such as the Swiss Catalogue of Learning Objectives [5], the Canadian CANMEDS acting model [6], the Dutch Blueprint [7] and Scottish learn-goal catalog [8], all that are being used as guiding principles in various fields of medical education and further training and are continually evolving [9].

In German-speaking countries, Skills-Labs have been founded at medical faculties since the nineties to live up to the increased requirements of this sector [10]. The changes in the approbation regulation of 2002 [https://www.gesetze-im-internet.de/_appro_2002/ cited 13.07.2016], the initiation of the tuition fees in many German states in 2007 and the initiation of a national practical exam in Switzerland in 2011 [11] supported this development at the faculties to exert themselves more for the practical and communicative skills of their graduates and to establish supportive Skills-Labs [12].

The GMA-committee Practical Skills [https://gesellschaft-medizinische-ausbildung.org/ausschuesse/praktische-fertigkeiten.html cited 13.07.2016] was founded in 2007 to foster the mediation of practical skills and to strengthen the research on this topic [https://gesellschaft-medizinische-ausbildung.org/ausschuesse/praktische-fertigkeiten.html cited 13.07.2016].

It is also worth mentioning that these activities and the caused paradigm shift in the medical education, have also lead to the event of deciding the National competence-based learning-goal catalog (NKLM) [http://www.nklm.de/kataloge/nklm/lernziel/uebersicht cited 13.07.2016], [13] and the national learning-goal catalog dentistry (NKLZ) [http://www.nklz.de/kataloge/nklm/lernziel/uebersicht cited 13.07.2016] by a large majority (29:3 votes, one abstention) on the medical faculty day on the fourth of July 2015 in Kiel. Basis of this chapter 14b (clinical-practical skills) was the, by the GMA-committee for practical skills developed, consensus statement practical skills [14]. 

These twenty-year-old developments deserve it to release a special edition on the topic of the “GMA-committee for practical skills”.

In this booklet, you can find the original work of different general topics of the mediation of practical skills, project reports and statements of the GMA-committee for exams, veterinary medicine and dentistry as to the GMA-committee for practical skills adjacent committees partly assigned with overlapping contents. In the beginning, there are comprehensive papers [15], [16], [17], [18], the following articles are the original papers picking up the recent scientific questions [19], [20], [21], [22], [23], [24], [25], [26], [27], [28], [29]. In the end they are followed by statements of the “adjacent” committees [30], [31], [32].

We would like to thank all the contributors in the field of practical skills, especially the authors and reviewers of the articles in this special edition and we wish the readers an inspiring read.

## Notes

Dedicated to the Pioneers who committed themselves to the teachings of practical skills during and after medical education

## Comepting interests

The authors declare that they have no competing interests.
